# Loop-Mediated Isothermal Amplification for Detecting Four Major Foodborne Pathogens in Meat and Meat Products

**DOI:** 10.3390/foods14132321

**Published:** 2025-06-30

**Authors:** Xin Li, Mingxue Zhu, Siyuan Wang, Weijia Li, Baohong Ren, Lingbo Qu, Xiaoling Zhang

**Affiliations:** 1 School of Pharmaceutical Sciences, Zhengzhou University, Zhengzhou 450001, China; 2 Key Laboratory of Food Safety Quick Testing and Smart Supervision Technology for State Market Regulation, Henan Province Food Inspection Research Institute, Zhengzhou 450003, China; 3 State Key Laboratory of Cotton Bio-Breeding and Integrated Utilization, Zhengzhou University, Zhengzhou 450001, China; 4 Zhengzhou Zhongdao Biotechnology Co., Ltd., Zhengzhou 450007, China; 5 Institute of Chemistry, Henan Academy of Sciences, Zhengzhou 450002, China

**Keywords:** loop-mediated isothermal amplification, meat and meat products, foodborne pathogenic bacteria, visualization, on-site rapid detection

## Abstract

*Listeria monocytogenes*, *Staphylococcus aureus*, *Salmonella enterica*, and *Escherichia coli* O157:H7 are four major foodborne pathogenic bacteria found in meat and meat products, which pose significant threats to human health. In this study, we developed specific loop-mediated isothermal amplification (LAMP) primers targeting these four pathogenic bacteria. Following the optimization of system components and reaction parameters, four rapid and simplified LAMP-based detection assays were established, which enabled the visual detection of these four pathogenic bacteria within 40–50 min. The three established LAMP assays targeting *L. monocytogenes*, *S. aureus*, and *E. coli* O157:H7 achieved species-level discrimination, whereas the LAMP method for *Salmonella* exhibited genus-level specificity. The detection limits of the LAMP assays were determined as follows: 1.8 × 10^1^ colony forming units (CFU)/mL for *L. monocytogenes*, 5.1 × 10^1^ CFU/mL for *S. aureus*, 1.2 × 10^1^ CFU/mL for *S. enterica*, and 3.3 × 10^3^ CFU/mL for *E. coli* O157:H7, with sensitivity improved by 10–1000-fold compared to conventional PCR. The developed LAMP assays were used to analyze 52 meat and meat product samples, and 7 samples were positive, which was consistent with the results of the conventional PCR and culture-based methods, demonstrating an accuracy rate of 100% for the LAMP methods. In conclusion, the established LAMP assays exhibit high specificity, enhanced sensitivity, and result visualization, making them suitable for on-site rapid detection in food safety monitoring.

## 1. Introduction

Meat and meat products provide high-quality protein, vitamins, and other nutrients in human diets [1]. The high nutrient content and moisture levels in meat and meat products create ideal conditions for the rapid proliferation of microorganisms and foodborne pathogens, leading to severe economic losses and health hazards [2]. Common foodborne pathogenic bacteria mainly include *Listeria monocytogenes*, *Staphylococcus aureus*, *Salmonella*, and *Escherichia coli*, and food products contaminated by these pathogens pose many disease risks to millions of consumers annually [3].

*L. monocytogenes* can survive under a wide range of temperatures, broad pH levels, and high salt concentrations, as well as other adverse conditions. It can adapt to and even grow in diverse food production environments [4]. Listeriosis is a rare, preventable, and treatable foodborne disease, but has high hospitalization and high case fatality rates of 20–50%, presenting as gastroenteritis, septicemia, central nervous system (CNS) infections, and maternal–fetal infections [5]. *S. aureus* lives in different niches, including the skin, nares, and mucosal surfaces of more than one-third of the human population [6]. *S. aureus* commonly causes staphylococcal food poisoning and other clinical syndromes through the ingestion of heat-stable staphylococcal enterotoxins preformed in food [7]. *Salmonella* predominantly resides in the intestinal tract of humans and animals [8]. *Salmonella* transmission to humans happens along the farm-to-fork continuum via contaminated animal- and plant-derived foods [9]. Infections caused by *Salmonella* can lead to various illnesses, including gastroenteritis, bacteremia, and septicemia [8]. *E. coli* O157:H7 is the most common serotype of *E. coli* and specifically colonizes the large intestine [10], causing acute gastroenteritis, hemorrhagic colitis, and hemolytic uremic syndrome in humans [11]. These four foodborne pathogenic bacteria can cause rapid infection, resulting in significant risks to public health and a substantial economic burden worldwide [12]. Therefore, the development of rapid, sensitive, and accurate detection methods for foodborne pathogenic bacteria is one of the key strategies to ensure food safety.

Currently, a variety of methods have been developed to detect foodborne pathogens to protect public health, such as culture-based methods, immunological assays, and nucleic-acid-based methods [13]. Culture-based methods remain the reference methods, which use genus-specific selective media to isolate and count viable colonies of foodborne pathogens, but they always require up to three days to cultivate bacteria and exhibit a relatively low detection accuracy [14]. Immunological assays can rapidly and accurately detect foodborne pathogens based on the specific binding between microbial antigens and antibodies, but their detection sensitivity is often not high [15]. Nucleic acid-based methods, such as Polymerase Chain Reaction (PCR), quantitative PCR, and Reverse Transcriptase PCR (RT-PCR), based on the use of primers targeting the DNA of these pathogens, are fast (3–6 h) and sensitive, but they require complicated nucleic acid extraction procedures and expensive equipment [15,16]. Loop-mediated isothermal amplification (LAMP) is a molecular diagnostic technology enabling rapid nucleic acid amplification at a constant temperature [17]. LAMP employs from four to six primers targeting from six to eight regions of the target gene sequence, allowing for amplification at temperatures between 60 and 65 °C and the production of up to 10^9^ copies within 1 h, compared to 10^6^ copies yielded by PCR [17,18]. LAMP’s isothermal nature eliminates the need for advanced thermal cyclers, employing a simple heating block to maintain a constant temperature, thus facilitating on-site rapid testing [19]. Hydroxynaphthol blue (HNB) is a frequently used metal indicator in LAMP assays. HNB binds to Mg^2+^ ions and turns violet in a negative sample, however, in a positive sample, as the LAMP reaction proceeds, the generation of pyrophosphate depletes Mg^2+^ ions, causing the indicator to turn sky blue [20]. The addition of HNB to the LAMP reaction system enables visual detection through the direct naked-eye observation of color changes before and after amplification.

This study developed four visual LAMP-based assays using HNB as a colorimetric indicator for the rapid detection of four common foodborne pathogens in meat and meat products. By optimizing experimental parameters such as Mg^2+^ concentrations, primer ratios, and reaction temperatures and time, pathogen-specific detection systems were developed. The specificity and sensitivity of the optimized systems were rigorously validated through cross-reactivity tests with non-target microorganisms and serial dilutions of pathogen DNA. Furthermore, the method demonstrated a 100% accuracy in practical applications when tested on 52 meat and meat product samples, confirming its reliability for on-site rapid detection. This approach provides a practical reference for rapid pathogen detection in meat processing and quality control.

## 2. Materials and Methods

### 2.1. Materials and Reagents

The experimental strains included *Listeria monocytogenes* (ATCC 15313), *Staphylococcus aureus* (ATCC 29213), *Staphylococcus epidermidis* (LJJ-2), *Staphylococcus capitis* (YP-1), *Staphylococcus xylosus* (JHGL-3), *Salmonella enterica* (CICC 21484), *Escherichia fergusonii* (LSD6-1), *Escherichia coli* (LSD1-5), *Shigella flexneri* (LSD5-3), *Shigella sonnei* (LZ-5), *Bacillus cereus* (LHH-3), and *Pseudomonas aeruginosa* (DST-11), all preserved by our laboratory. Escherichia coli O157:H7 (ATCC 43895), *Listeria ivanovii* (CICC21633), and *Listeria innocua* (ATCC7830) were provided by Zhengzhou Zhongdao Biotechnology Co., Ltd. (Zhengzhou, China) for free. *Salmonella bongori* (BNCC366367) was bought from Shangcheng Beinachuanglian Biotechnology Co., Ltd. (Xinyang, China).

The Bacterial Genomic DNA Extraction Kit (D1600-100) was bought from Beijing Solarbio Science & Technology Co., Ltd. (Beijing, China). Bst DNA Polymerase Large Fragment (8 U/mL), dNTP Mix (10 mM), MgSO_4_ (100 mM), 10× ThermoPol Buffer, and 6× DNA Loading Buffer were obtained from Nanjing Vazyme Biotech Co., Ltd. (Jiangsu, China). HNB (H103107) was purchased from Shanghai Aladdin Biochemical Technology Co., Ltd. (Shanghai, China). High-purity low-electroendosmosis agarose (TSJ001) was purchased from Beijing Tsingke Biotechnology Co., Ltd. (Beijing, China). DS2000 DNA Marker (M1101) was bought from Guangzhou Dongsheng Biotechnology Co., Ltd. (Guangzhou, China). ExRed nucleic acid electrophoresis dye (ZS203-1) was purchased from Beijing Zhuangmeng International Bio-Gene Technology Co., Ltd. (Beijing, China). Tryptone (01-202), Yeast Extract Powder (01-012), Agar Powder (01-023), and Sodium Chloride of LB medium were bought from Beijing Aoboxing Biotechnology Co., Ltd. (Beijing, China). The DK-8D electric-heated thermostatic water bath was bought from Shanghai Yiheng Technology Co., Ltd. (Shanghai, China). The ABI Veriti 96 PCR system was purchased from Thermo Fisher Scientific (Waltham, MA, USA). The 721 visible spectrophotometer was produced by Shanghai Youke Instrument Co., Ltd. (Shanghai, China). The electrophoresis apparatus was bought from Bio-Rad (Hercules, CA, USA).

### 2.2. Bacterial Recovery Culture and Genomic DNA Extraction

Sixteen strains (*L. monocytogenes*, *S. aureus*, *S. enterica*, *E. coli* O157:H7, *E. fergusonii*, *S. flexneri*, *S. sonnei*, *B. cereus*, *E. coli*, *P. aeruginosa*, *S. epidermidis*, *S. capitis*, *S. xylosus*, *L. ivanovii*, *L. innocua*, and *S. bongori*) were taken out from a −80 °C freezer and thawed at room temperature. Then, after resuscitation by four-zone streaking on LB solid medium, the plates were inverted and incubated at 37 °C for 18–24 h. After single colonies appeared, individual colonies were transferred into fresh LB liquid medium and incubated at 37 °C with shaking at 220 rpm for 16–24 h. In total, 1 mL of bacterial suspension was used to extract the genomic DNA of each strain following the instructions of Bacterial Genomic DNA Extraction Kit. The concentration and purity of the DNA templates were determined and stored at −20 °C for downstream applications.

### 2.3. LAMP and PCR Primer Design

The *prfA* gene (GenBank ID: NC003210.1) of *L. monocytogenes* [21], the *nuc* gene (GenBank ID: DQ507379.1) of *S. aureus* [22], the *slyA* gene (GenBank ID: NC003197.2) of *S. enterica* [23], and the *rfbE* gene (GenBank ID: S83460.1) of *E. coli* O157:H7 [24] were selected as target genes, and their highly conserved sequences were identified using BLASTN (v2.14.1+) in NCBI. The LAMP primers were designed using Primer Explorer V5 software (available online: https://primerexplorer.eiken.co.jp/lampv5e/index.html, accessed on 12 July 2024). After preliminary experiments, one set of LAMP primers was selected for each gene (Table 1). PCR primers were also designed for each gene used for conventional PCR detection (Table 1). All primers were synthesized by Beijing Tsingke Biotechnology Co., Ltd. (Beijing, China).

### 2.4. Optimization of LAMP Reaction Systems

The LAMP amplification system contained 10× ThermPol buffer, MgSO_4_, dNTP Mix, outer primers (B3 and F3), inner primers (FIP and BIP), Bst DNA polymerase, HNB, and template DNA. Single-variable optimization was performed for the LAMP reaction system, separately targeting four foodborne pathogens. The following seven parameters were optimized sequentially: Mg^2+^ concentration (2 mM, 4 mM, 6 mM, 8 mM, 10 mM, and 12 mM), dNTP Mix volume (2.0 μL, 2.5 μL, 3.0 μL, 3.5 μL, 4.0 μL, and 4.5 μL), inner-to-outer primer ratio (1:1, 2:1, 4:1, 8:1, 16:1, and 32:1), Bst DNA polymerase volume (0.4 μL, 0.6 μL, 0.8 μL, 1.0 μL, 1.2 μL, and 1.4 μL), reaction temperature (60 °C, 61 °C, 62 °C, 63 °C, 64 °C, and 65 °C), reaction duration (10 min, 20 min, 30 min, 40 min, 50 min, and 60 min), and HNB concentration (60 μM, 90 μM, 120 μM, and 150 μM). The optimal reaction parameters were determined through systematic comparison.

### 2.5. Specificity Assessment

Twelve different strains (*E. fergusonii*, *S. flexneri*, *S. sonnei*, *B. cereus*, *E. coli*, *P. aeruginosa*, *S. epidermidis*, *S. capitis*, *S. xylosus*, *L. ivanovii*., *L. innocua*, and *S. bongori*) and four foodborne pathogenic bacteria (*L. monocytogenes*, *S. aureus*, *S. enterica*, and *E. coli* O157:H7) were simultaneously used to test the specificity of the previously established LAMP amplification systems.

### 2.6. Sensitivity Assessment

The bacterial cultures (*L. monocytogenes*, *S. aureus*, *S. enterica*, and *E. coli* O157:H7) in the logarithmic growth phase (OD_600_ = 0.6–0.8) were quantified via the dilution plate counting method. Serial ten-fold dilutions with 0.9% NaCl generated eight concentration gradients. Genomic DNA from the diluted samples was extracted separately as templates to assess the detection limits of the optimized LAMP assays and conventional PCR. By comparing the detection limit values, the detection sensitivities of the two methods were compared.

### 2.7. PCR Reaction System of Four Foodborne Pathogenic Bacteria

The total volume of 20 μL in the PCR reaction system included 1.0 μL of genomic DNA template, 1.0 μL of forward primer, 1.0 μL of reverse primer, 10 μL of 2 × Magic Green Taq SuperMix, and 7.0 μL of ddH_2_O. The PCR protocols were performed as follows: pre-denaturation at 95 °C for 3 min, followed by 27 cycles of denaturation at 95 °C for 10 s, annealing at 55 °C for 10 s, and extension at 72 °C, with varying durations corresponding to different pathogenic bacteria (43 s for *L. monocytogenes*, 39 s for *S. aureus*, 27 s for *S. enterica*, and 66 s for *E. coli* O157:H7). A final extension at 72 °C for 5 min was applied to ensure the complete synthesis of all amplicons. Then, 1% agarose gel electrophoresis was performed to detect the PCR amplification results.

### 2.8. Detection of Four Foodborne Pathogenic Bacteria in Meat and Meat Products

To evaluate the practical application performance of the optimized LAMP reaction system, this study collected 52 meat and meat product samples from multiple meat food purchasing places in the high-tech zone of Zhengzhou City, Henan Province, China, including supermarkets, mini-markets, farmers’ markets, and mobile vendors, where local residents routinely buy meat foods. The samples consisted of 27 raw meats, 22 cooked meat products, 1 sausage, 1 hotpot meatball, and 1 quick-frozen dumpling, representing typical meat foods consumed daily. The optimized LAMP assays and conventional PCR methods were used to detect the four foodborne pathogenic bacteria in each sample. The accuracy of the LAMP detection method for each pathogenic bacteria was calculated by comparing the results with those of the PCR and culture-based methods. The detected positive samples were further confirmed using culture-based methods. Sterile water was used as a negative control, and pathogenic bacterial genomic DNA was used as a positive control in the LAMP and PCR experiments.

The sample processing procedure was conducted as follows: Sterile cotton swabs moistened with PBS were used to swab the sample surfaces. The swabs were then placed in 1.5-mL centrifuge tubes containing 1 mL of PBS, vortexed for 1 min, and subsequently removed. After capping the tubes, centrifugation was performed at 12,000 rpm for 1 min, followed by supernatant removal. The pellet was washed once with 1 mL of PBS (12,000 rpm, 1 min centrifugation), and the supernatant was discarded. The resulting pellet was resuspended in 70 μL of ddH_2_O, subjected to a 10 min boiling water bath, and centrifuged again at 12,000 rpm for 1 min. The supernatant was collected as the genomic DNA template for LAMP and PCR analysis.

### 2.9. Culture-Based Methods for Salmonella and E. coli O157:H7

LAMP-positive meat food samples underwent further confirmation by culture-based methods. For *Salmonella* confirmation, 5 g of positive sample was transferred into 50 mL of buffered peptone water (BPW) and incubated at 37 °C for 12 h for pre-enrichment. Subsequently, 1 mL of BPW culture was inoculated into 10 mL of tetrathionate brilliant green enrichment broth (TTB) and incubated at 37 °C for 12 h for selective enrichment. A loopful of TTB culture was streaked onto a bismuth sulfite (BS) agar plate and incubated at 37 °C for 40–48 h [25]. The colonies of *Salmonella* appeared black with a metallic sheen, brown, or gray, with the surrounding medium exhibiting black or brown discoloration. The growth of negative bacteria was inhibited, or the colonies appeared black lacking a metallic luster.

For *E. coli* O157:H7 confirmation, 5 g of positive sample was transferred into 50 mL of Modified *E. coli* Broth with novobiocin and incubated at 37 °C for 18 h. Then, a loopful of the enriched culture was streaked onto Sorbitol MacConkey Agar (SMAC) plates and incubated at 37 °C for 18–24 h [26]. The colonies of *E. coli* O157:H7 appeared as colorless, flat, and transparent, with smooth edges and a pale brown central region. Other negative bacteria mostly formed pink colonies or failed to grow.

## 3. Results and Discussion

### 3.1. Optimization of LAMP Reactions

The LAMP system components and reaction parameters were systematically optimized, which included the Mg^2+^ concentration, Bst DNA polymerase volume, HNB concentration, and reaction temperature and time, with additional factors detailed in the Supplementary Materials (Figures S1–S7). Notably, the pre-incorporation of HNB into our LAMP protocol eliminated contamination risks associated with post-reaction dye supplementation (like SYBR Green), which inherently introduces aerosol contamination through tube-opening steps [27]. The optimized LAMP systems for foodborne pathogenic bacteria are shown in Table 2. Visual detection is achievable by LAMP amplification at 64 °C for 50 min for *L. monocytogenes*, 62 °C for 40 min for *S. aureus* and *S. enterica*, and 61 °C for 50 min for *E. coli* O157:H7.

### 3.2. Specificity of the Established LAMP Assays

**Figure 1:**
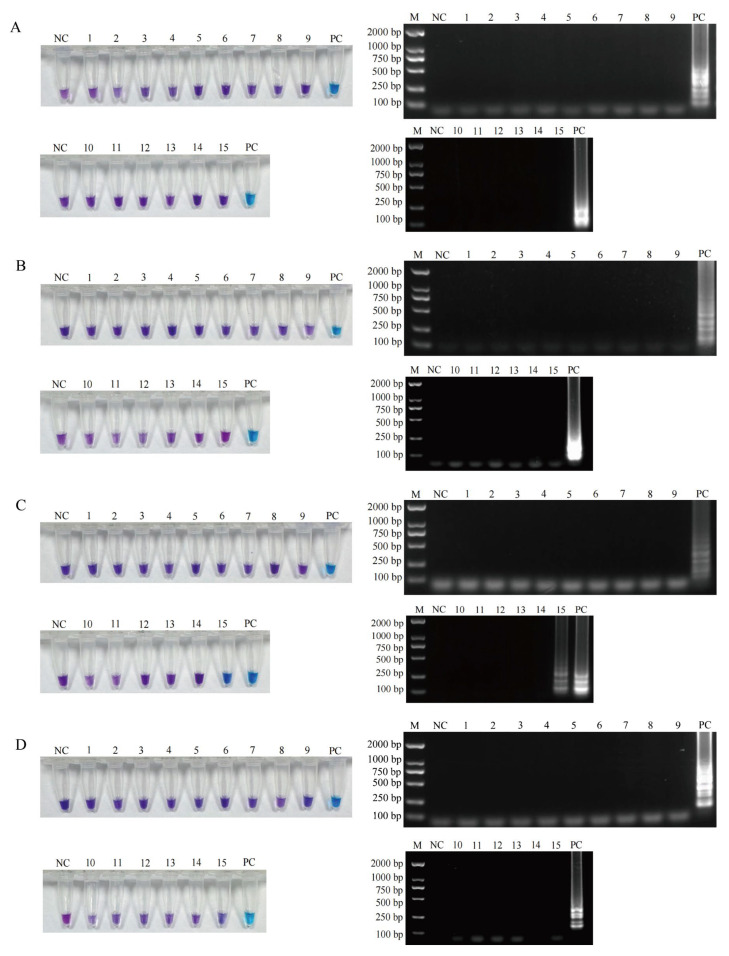
Specificity analysis of the four established LAMP assays was individually validated for each target foodborne pathogenic bacteria. (**A**) The specificity results of the LAMP assay for *L. monocytogenes*, samples 1–3 are *S. aureus*, *S. enterica*, and *E. coli* O157:H7, PC is *L. monocytogenes*. (**B**) the specificity results of the LAMP assay for *S. aureus*, samples 1–3 are *L. monocytogenes*, *S. enterica*, and *E. coli* O157:H7, PC is *S. aureus*. (**C**) the specificity results of the LAMP assay for *S. enterica*, samples 1–3 are *L. monocytogenes*, *S. aureus*, and *E. coli* O157:H7, PC is *S. enterica*. (**D**) the specificity results of the LAMP assay for *E. coli* O157:H7, samples 1–3 are *L. monocytogenes*, *S. aureus*, and *S. enterica*, PC is *E. coli* O157:H7. Every NC is sterile water as a negative control, and all samples 4–15 are *E. fergusonii*, *S. flexneri*, *S. sonnei*, *B. cereus*, *E. coli*, *P. aeruginosa, S. xylosus*, *S. epidermidis*, *S. capitis*, *L. innocua*, *L. ivanovii*, and *S. bongori*. M: DS2000 DNA marker. Violet: negative reaction, sky blue: positive reaction.

Sixteen bacteria, including the four target foodborne pathogens and twelve other bacteria, were used to evaluate the specificity of the established LAMP assays. For the LAMP detection systems of *L. monocytogenes*, *S. aureus*, and *E. coli* O157:H7, only tubes containing the target pathogens exhibited sky blue and the corresponding amplification bands, while all other fifteen bacteria tubes and the negative control sterile water tube remained violet with no amplification bands (Figure 1A,B,D). Thus, the three established LAMP assays achieved species-level discrimination. Both the *S. enterica* and *S. bongori* tubes showed positive results (Figure 1C), suggesting that the LAMP method for *Salmonella* exhibited genus-level specificity.

### 3.3. Sensitivity of the Established LAMP Methods

The counts of the four foodborne pathogenic pure cultures in the logarithmic growth phase (OD_600_ = 0.6–0.8) were determined using the dilution plate method, and the concentrations of *L. monocytogenes*, *S. aureus*, *S. enterica*, and *E. coli* O157:H7 were quantified as 1.8 × 10^9^ CFU/mL, 5.1 × 10^9^ CFU/mL, 1.2 × 10^9^ CFU/mL, and 3.3 × 10^9^ CFU/mL, respectively. Each pathogenic bacterium sample was subjected to 10-fold serial dilution to obtain eight concentration gradients, then all samples were detected by LAMP and PCR simultaneously. The detection limits of the LAMP assays were determined as follows: 1.8 × 10^1^ CFU/mL for *L. monocytogenes*, 5.1 × 10^1^ CFU/mL for *S. aureus*, 1.2 × 10^1^ CFU/mL for *S. enterica*, and 3.3 × 10^3^ CFU/mL for *E. coli* O157:H7, which were 100-fold lower than those of PCR for *L. monocytogenes* and *S. aureus*, 1000-fold lower for *S. enterica*, and 10-fold lower for *E. coli* O157:H7. These results demonstrate that the LAMP assays exhibited 10- to 1000-fold higher sensitivity than conventional PCR for detecting these four foodborne pathogens (Table 3). Moreover, the detection limits of *L. monocytogenes*, *S. aureus*, and *S. enterica* detected by our LAMP systems were in the same order of magnitude as those reported in other existing research with LAMP or LAMP-derivative techniques [28,29,30], confirming the enhanced sensitivity of the established LAMP methods.

### 3.4. Detection Performance of the Established LAMP Methods in Meat and Meat Product Samples

**Figure 2:**
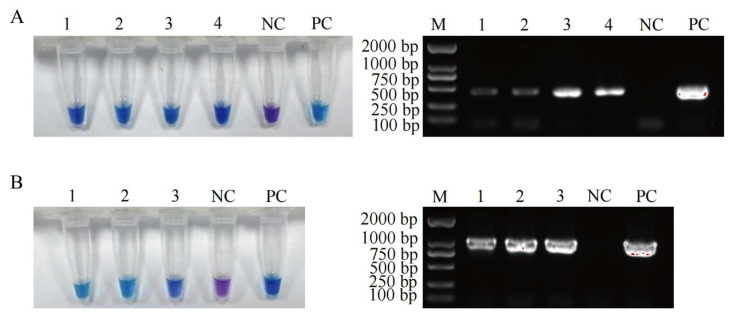
Specificity. The detection results of positive samples by LAMP and PCR assays. (**A**) The detection results of 4 *S. enterica* positive samples by LAMP and PCR assays, sample 1: raw chicken thigh meat, sample 2: raw beef, sample 3: raw pork, sample 4: raw duck thigh meat, NC: sterile water as negative control, PC: *S. enterica* as positive control. (**B**) The detection results of 3 *E. coli* O157:H7 positive samples by LAMP and PCR assays, sample 1: marinated pork head meat, sample 2: marinated chicken legs, sample 3: marinated pig trotters, NC: sterile water as negative control, PC: *E. coli* O157:H7 as positive control. M: DS2000 DNA marker. Violet: negative reaction, sky blue: positive reaction.

The contamination status of four foodborne pathogenic bacteria in 52 samples was analyzed using both the established LAMP detection methods and PCR methods. The results showed that 4 *S. enterica*-positive samples and 3 *E. coli* O157:H7-positive samples were detected by LAMP, and the PCR detection results were consistent with these findings (Figure 2). The contamination of pathogenic bacteria in the seven positive samples was further verified by culture-based methods (Figures S8 and S9). These results demonstrate that the accuracies of the designed LAMP methods for detecting *L. monocytogenes*, *S. aureus*, *S. enterica*, and *E. coli* O157:H7 were 100% (Table 4). Given the limited sample size (*n* = 52), broader sampling and the inclusion of additional sample types remain necessary to further confirm the accuracy of the developed LAMP methods.

The four *S. enterica*-positive samples were raw meats purchased from farmers’ markets or mini-markets, including chicken thigh meat, beef, pork, and duck thigh meat. The detection rate for *Salmonella* in raw livestock meat was 10.54%, while in pork, it reached 17.60% in 2515 raw livestock meat samples collected across 15 provinces in China during 2021 [31]. These results indicate that *Salmonella* contamination is highly prevalent in raw meat, so enhanced detection and control strategies for *Salmonella* are urgent. The three *E. coli* O157:H7-positive samples were cooked meat products bought from mobile vendors, which consisted of marinated pork head meat, marinated chicken legs, and marinated pig trotters. A previous study showed that 5-log reductions of *E. coli* O157:H7 were achieved in 6.54 min by cooking meatballs to an internal temperature of 85 °C [32]. Therefore, to minimize the risk of *E. coli* O157:H7 and other pathogens, cooked meat purchased from mobile vendors should be thoroughly reheated before consumption. Notably, none of the four pathogenic bacteria were detected in the 10 raw and 10 cooked meat samples purchased from supermarkets, which may be attributed to the faster turnover cycles and stricter quarantine procedures of meat and meat products sold in large supermarkets.

## 4. Conclusions

This study successfully established four optimized LAMP assays for the rapid detection of *L. monocytogenes*, *S. aureus*, *S. enterica*, and *E. coli* O157:H7, with the entire detection process completed within 60 min. Colorimetric changes were observed only for the target pathogens among the 16 tested pathogenic bacteria, demonstrating the high specificity of the LAMP methods. Comparative studies revealed that the LAMP assays exhibited 10-fold to 1000-fold higher sensitivity than conventional PCR for detecting these four foodborne pathogens. Furthermore, the incorporation of HNB enabled the visual interpretation of results without requiring specialized equipment, making the LAMP assays particularly suitable for on-site rapid testing in local food safety regulatory agencies. Practical validation using 52 meat and meat product samples achieved a 100% accuracy, confirming the reliability of the developed LAMP methods and highlighting their strong potential for food safety monitoring applications.

## Figures and Tables

**Table 1:** Nucleotide sequence of LAMP and PCR primers.

Gene	Primer Type	Primer Name	Sequence (5′ → 3′)
*prfA*	LAMP primer	LM-F3	GTATTAGCGAGAACGGGAC
		LM-B3	TTGTTTTTGTAGGGTTTGGAA
		LM-FIP	GCCAACCGATGTTTCTGTATCAATAATTTACAATACTACAAAGGGGCT
		LM-BIP	GCAGGCTACCGCATACGTTAAAAGTGCGTAAGATTTTTGCTC
	PCR primer	LM-F	ATGAACGCTCAAGCAGAAGAATTCAAA
		LM-R	TTAATTTAATTTTCCCCAAGTAGCAGGAC
*nuc*	LAMP primer	SA-F3	AACAGTATATAGTGCAACTTCAA
		SA-B3	CTTTGTCAAACTCGACTTCAA
		SA-FIP	ATGTCATTGGTTGACCTTTGTACATAAATTACATAAAGAACCTGCGA
		SA-BIP	GTTGATACACCTGAAACAAAGCATCATTTTTTTCGTAAATGCACTTGC
	PCR primer	SA-F	ATGACAGAATACTTATTAAGTGCTG
		SA-R	AAATATTTAATTTCTTTTTTTTCGCTTGTG
*slyA*	LAMP primer	SE-F3	TTCTGATCTGGCACGGTTG
		SE-B3	GATCCAACGTGCGTACCAG
		SE-FIP	TGACCCAATGTGTCTGCGTCAACATTTGGCGTGCTCTGATTG
		SE-BIP	TCATCAATTGCCGCCTGACCACTGCTCAATGCCTATCGCTT
	PCR primer	SE-F	AAGGAGATGAAATTGGAATCGCCA
		SE-R	TCAATCGTGAGAGTGCAATTCCATA
*rfbE*	LAMP primer	EC-F3	AGCGTTAGGTATATCGGAAG
		EC-B3	GTTCCATATCACATGGATGTC
		EC-FIP	TGGCTCCTGTGTATTTTATAGCATTGAGATGAAGTTATTGTTCCAACA
		EC-BIP	TGAAACTTGGCAAATGTCTGTTAGTAATGGACACACATAATAGCTTTA
	PCR primer	EC-F	ATGAAATATATACCAGTTTACCAACC
		EC-R	CTATTTATCACTATAAAATTCGTTAATAGA

**Table 2:** Optimized LAMP system components and reaction parameters.

Items	LAMP System for *L. monocytogenes*	LAMP System for *S. aureus*	LAMP System for *S. enterica*	LAMP System for *E. coli* O157:H7
10× ThermPol Buffer	2.5 μL	2.5 μL	2.5 μL	2.5 μL
MgSO_4_ (100 mM)	1.5 μL	1.5 μL	1.5 μL	1.5 μL
dNTP Mix (10 mM)	3.0 μL	2.5 μL	2.5 μL	3.0 μL
F3/B3 primers (10 µM)	0.5 μL	0.5 μL	0.5 μL	0.5 μL
FIP/BIP primers (10 µM)	4.0 μL	4.0 μL	4.0 μL	4.0 μL
Bst DNA polymerase	0.4 μL	0.8 μL	0.6 μL	0.8 μL
HNB (3 mM)	1.0 μL	0.75 μL	0.75 μL	0.75 μL
Genomic DNA template	100 ng	100 ng	100 ng	100 ng
ddH_2_O	Fill up to 25 μL	Fill up to 25 μL	Fill up to 25 μL	Fill up to 25 μL
Reaction temperature	64 °C	62 °C	62 °C	61 °C
Reaction time	50 min	40 min	40 min	50 min

**Table 3:** Comparison of the sensitivity of LAMP and conventional PCR for the detection of four pathogenic bacteria.

Strains	LAMP Detection Limit (CFU/mL)	PCR Detection Limit (CFU/mL)	Fold Change
*L. monocytogenes*	1.8 × 10^1^	1.8 × 10^3^	100
*S. aureus*	5.1 × 10^1^	5.1 × 10^3^	100
*S. enterica*	1.2 × 10^1^	1.2 × 10^4^	1000
*E. coli* O157:H7	3.3 × 10^3^	3.3 × 10^4^	10

**Table 4:** The detection results of 52 samples.

Strains	Total Samples	LAMP Detection		Accuracy Rate
		Positive Samples	Negative Samples	
*L. monocytogenes*	52	0	52	100%
*S. aureus*	52	0	52	100%
*S. enterica*	52	4	48	100%
*E. coli* O157:H7	52	3	49	100%

## Data Availability

All data generated or analyzed during this study are included in this published article and its Supplementary Materials files.

## Supplementary Materials

The following supporting information can be downloaded at: https://www.mdpi.com/article/10.3390/foods14132321/s1, Figure S1: Optimization results of Mg^2+^ concentration for LAMP detection of four pathogenic bacteria. Figure S2: Optimization results of Bst DNA polymerase volume for LAMP detection of four pathogenic bacteria. Figure S3: Optimization results of dNTP Mix volume for LAMP detection of four pathogenic bacteria. Figure S4: Optimization results of the addition ratio of internal and external primers for LAMP detection of four pathogenic bacteria. Figure S5: Optimization results of reaction temperatures for LAMP detection of four pathogenic bacteria. Figure S6: Optimization results of reaction time for LAMP detection of four pathogenic bacteria. Figure S7: Optimization results of added concentration of HNB for LAMP detection of four pathogenic bacteria. Figure S8: Microbial culture results confirming *Salmonella* positive samples. Figure S9: Microbial culture results confirming *E. coli* O157:H7 positive samples.
