# Supplementary material for: Loop-Mediated Isothermal Amplification for Detecting Four Major Foodborne Pathogens in Meat and Meat Products
Source: Foods. 2025 Jun 30;14(13):2321. doi: 10.3390/foods14132321 (PMC12249438; doi:10.3390/foods14132321)
Supplement: Supplementary file 1 [file foods-14-02321-s001.zip › foods-3675305-supplementary.pdf]

Supplementary Materials for

Article

Loop-mediated isothermal amplification for detecting four major foodborne pathogens in meat and meat products

Xin Li [1,2,3], Mingxue Zhu [1], Siyuan Wang [1], Weijia Li [1], Baohong Ren [4], Lingbo Qu [2,3,5*] and Xiaoling Zhang [1,2,3*]

[1] School of Pharmaceutical Sciences, Zhengzhou University, Zhengzhou 450001, China

[2] Key Laboratory of Food Safety Quick Testing and Smart Supervision Technology for State Market Regulation, Henan Province Food Inspection Research Institute, Zhengzhou 450003, China

[3] State Key Laboratory of Cotton Bio-Breeding and Integrated Utilization, Zhengzhou University, Zhengzhou 450001, China

[4] Zhengzhou Zhongdao Biotechnology Co., Ltd., Zhengzhou 450007, China

[5] Institute of Chemistry, Henan Academy of Sciences, Zhengzhou 450000, China

* Correspondence: qulingbo@zzu.edu.cn (L.Q.); xiaolingzhang@zzu.edu.cn (X.Z.)

1. Optimization of Mg^{2+} Concentration

According to the agarose gel electrophoresis results, the condition with the brightest band and the lowest Mg^{2+} concentration was selected as the optimal Mg^{2+} concentration for LAMP detection of each foodborne pathogen. The best amplification results were obtained with the addition of 6 mM of MgSO4 for *L. monocytogenes*, *S. aureus*, *S. enterica*, and *E. coli* O157:H7 (Figure S1A-D).

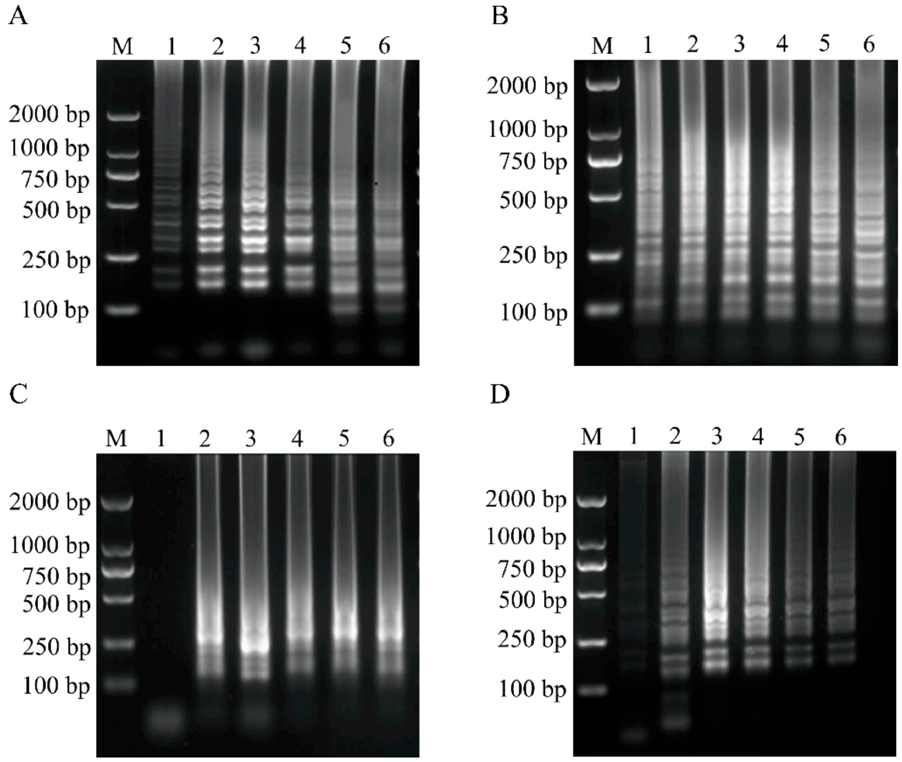

Figure S1. Optimization results of Mg^{2+} concentration for LAMP detection of four pathogenic bacteria. The agarose gel electrophoresis results of LAMP amplification performed on *L. monocytogenes* (A), *S. aureus* (B), *S. enterica* (C), and *E. coli* O157:H7 (D) under different Mg^{2+} concentrations. M: DS2000 DNA marker, lanes 1-6: Mg^{2+} concentrations at 2 mM, 4 mM, 6 mM, 8 mM, 10 mM, and 12 mM, respectively.

2. Optimization of Bst DNA Polymerase Addition

The amplification results obtained with different amounts of Bst DNA polymerase were compared, and the condition producing the brightest band while using the lowest enzyme concentration was selected as the optimal Bst DNA polymerase addition amount for LAMP detection of each foodborne pathogen. The

optimal Bst DNA polymerase volumes for LAMP detection were established as 0.4 μL for *L. monocytogenes* (Figure S2A), 0.8 μL for *S. aureus* (Figure S2B), 0.6 μL for *S. enterica* (Figure S2C), and 0.8 μL for *E. coli* O157:H7 (Figure S2D).

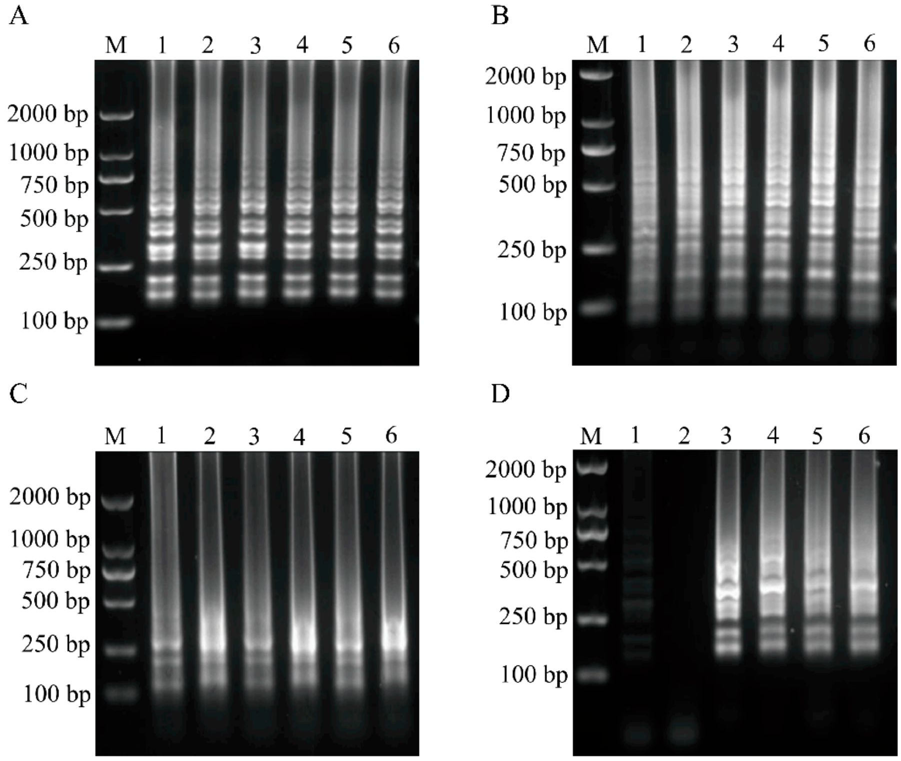

Figure S2. Optimization results of Bst DNA polymerase volume for LAMP detection of four pathogenic bacteria. The LAMP amplification results of *L. monocytogenes* (A), *S. aureus* (B), *S. enterica* (C), and *E. coli* O157:H7 (D) under varying Bst DNA polymerase volumes. M: DS2000 DNA marker, lanes 1-6: Bst DNA polymerase volumes 0.4 μL, 0.6 μL, 0.8 μL, 1.0 μL, 1.2 μL, and 1.4 μL, respectively.

3. Optimization of dNTP Mix Volume

Based on the agarose gel electrophoresis results of LAMP amplification with different volumes of dNTP Mix, the condition producing the brightest band with the lowest dNTP Mix volume was selected as the optimal dNTP Mix volume for LAMP detection of each foodborne pathogen. The optimal dNTP Mix volumes for LAMP detection were determined to be 3.0 μL for *L. monocytogenes* (Figure. S3A), 2.5 μL for *S. aureus* (Figure. S3B), 2.5 μL for *S. enterica* (Figure. S3C), and 3.0 μL for *E. coli* O157:H7 (Figure. S3D).

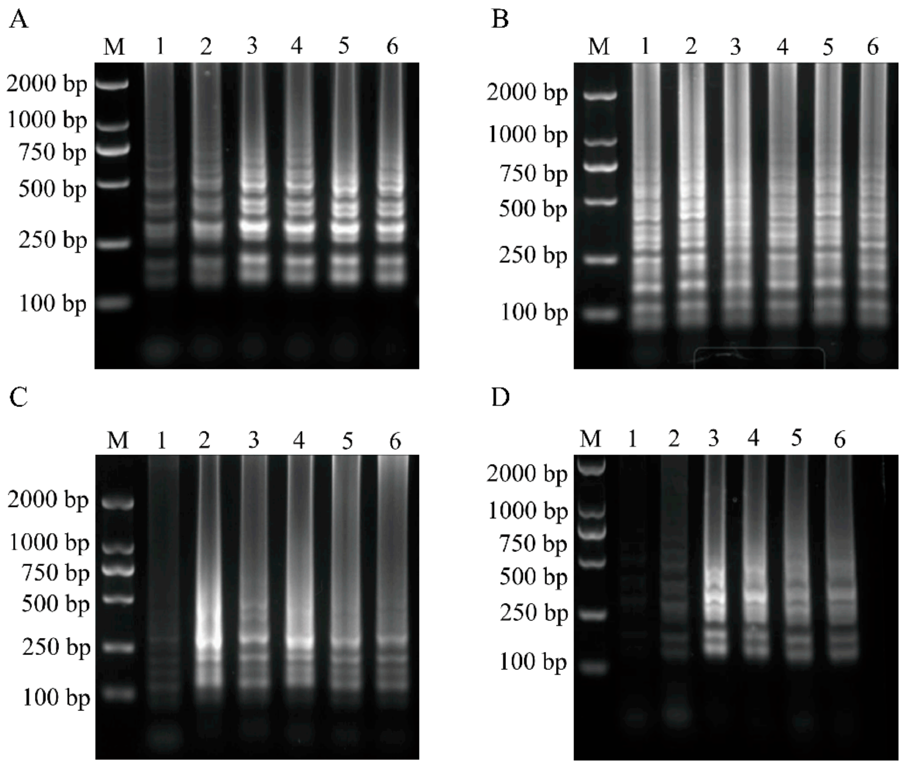

Figure S3. Optimization results of dNTP Mix volume for LAMP detection of four pathogenic bacteria. The LAMP amplification results of *L. monocytogenes* (A), *S. aureus* (B), *S. enterica* (C), and *E. coli* O157:H7 (D) under varying dNTP Mix volumes. M: DS2000 DNA marker, lanes 1-6: dNTP Mix volumes 2.0 μL, 2.5 μL, 3.0 μL, 3.5 μL, 4.0 μL, and 4.5 μL, respectively.

4. Optimization of the Ratio of Internal and External Primer Addition

The amplification results obtained with different ratios of internal and external primer concentrations were compared, and the condition showing the brightest band and minimum required dosage was selected as the optimal primer ratio for LAMP detection of each foodborne pathogen. The optimal internal and external primer ratios for LAMP detection of *L. monocytogenes*, *S. aureus*, *S. enterica*, and *E. coli* O157:H7 were confirmed as 8:1 (Figure S4A-D).

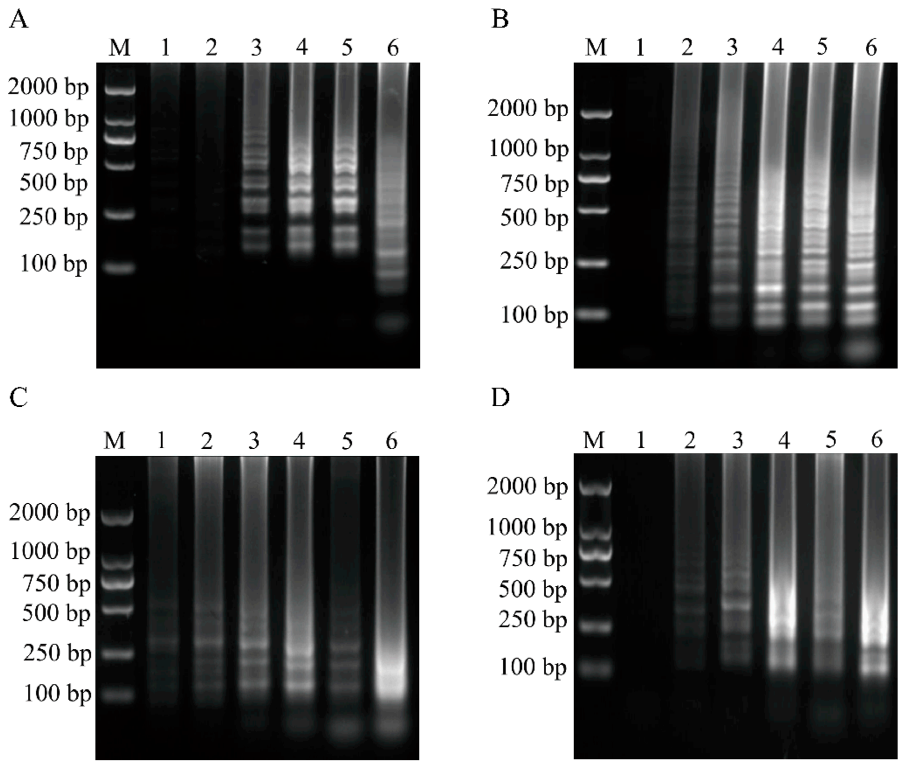

Figure S4. Optimization results of the addition ratio of internal and external primers for LAMP detection of four pathogenic bacteria. The LAMP amplification results of *L. monocytogenes* (A), *S. aureus* (B), *S. enterica* (C), and *E. coli* O157:H7 (D) using different ratios of internal and external primers. M: DS2000 DNA marker, lanes 1-6: the addition ratio of internal and external primers 1:1, 2:1, 4:1, 8:1, 16:1, and 32:1, respectively.

5. Optimization of Reaction Temperature

The amplification results at different reaction temperatures were analyzed by agarose gel electrophoresis, and the reaction temperature exhibiting the brightest amplification band was selected as the optimal temperature for LAMP detection of each foodborne pathogenic bacterium. The results showed that the optimal LAMP reaction temperature for *L. monocytogenes* was 64°C (Figure S5A), for *S. aureus* was 62°C (Figure S5B), for *S. enterica* was 62°C (Figure S5C), and for *E. coli* O157:H7 was 61°C (Figure S5D).

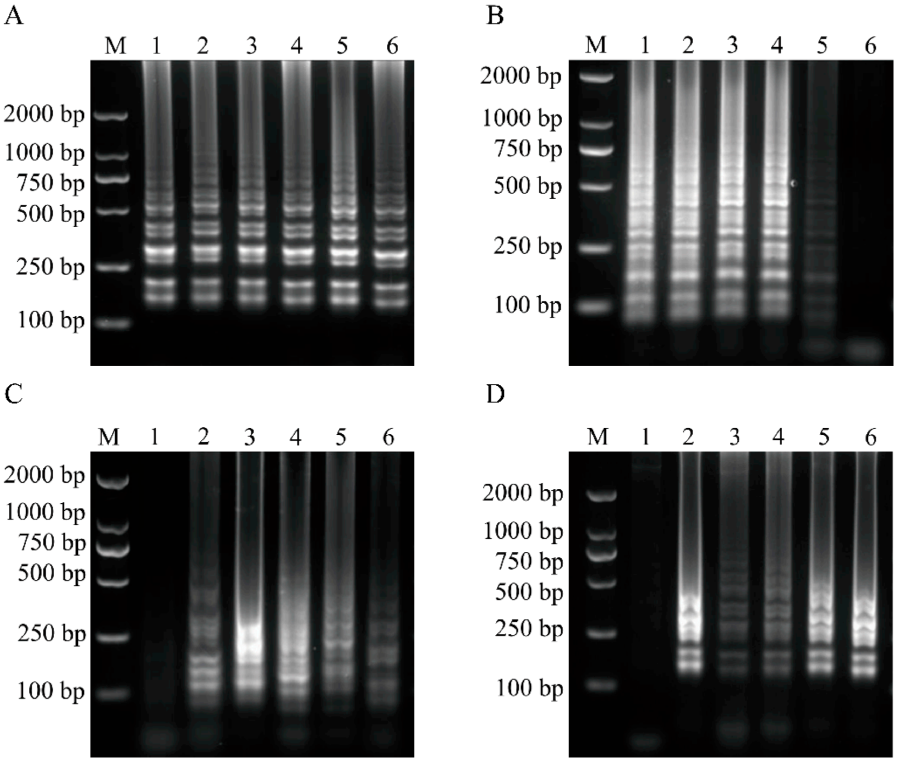

Figure S5. Optimization results of reaction temperatures for LAMP detection of four pathogenic bacteria. The agarose gel electrophoresis results of LAMP amplification performed on *L. monocytogenes* (A), *S. aureus* (B), *S. enterica* (C), and *E. coli* O157:H7 (D) at different temperatures. M: DS2000 DNA marker, lanes 1-6: LAMP amplification temperatures at 60°C, 61°C, 62°C, 63°C, 64°C, and 65°C, respectively.

6. Optimization of Reaction Time

Depending on the amplification results obtained under different reaction times, the condition exhibiting the brightest band with the shortest amplification duration was selected as the optimal reaction time for LAMP detection of each foodborne pathogen. The optimal reaction times for LAMP detection were determined as 50 min for *L. monocytogenes* (Figure S6A), 40 min for *S. aureus* (Figure S6B), 40 min for *S. enterica* (Figure S6C), and 50 min for *E. coli* O157:H7 (Figure S6D).

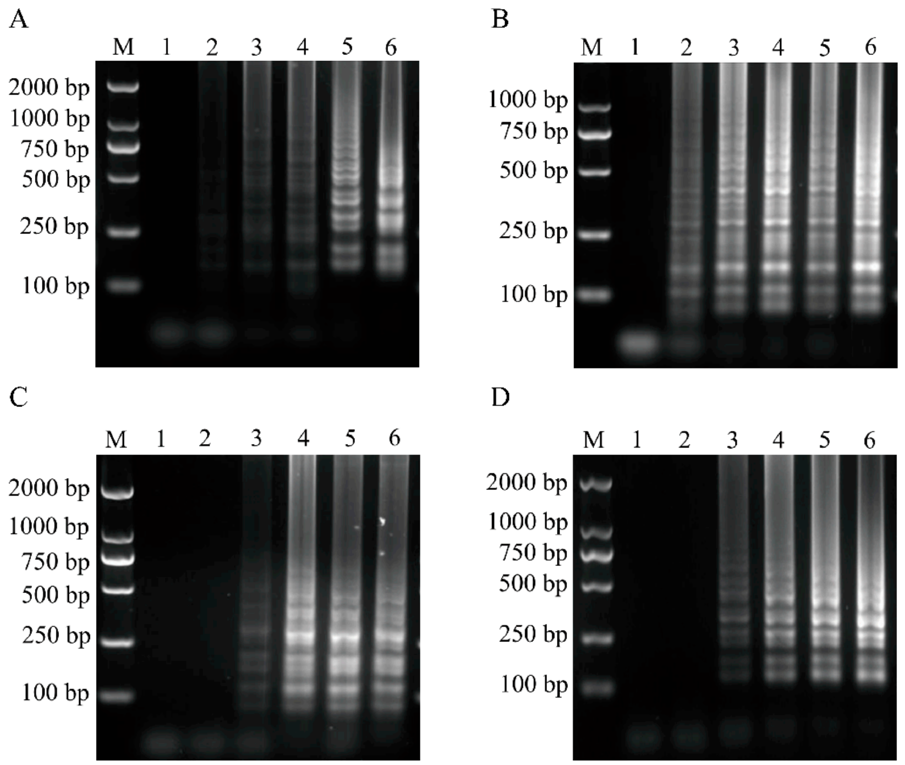

Figure S6. Optimization results of reaction time for LAMP detection of four pathogenic bacteria. The LAMP amplification results of *L. monocytogenes* (A), *S. aureus* (B), *S. enterica* (C), and *E. coli* O157:H7 (D) after different reaction times. M: DS2000 DNA marker, lanes 1-6: Reaction time 10 min, 20 min, 30 min, 40 min, 50 min, and 60 min, respectively.

7. Optimization of HNB Concentration

Based on visual observation, the lowest concentration that produced the most significant color difference between the negative and positive controls was chose as the optimal HNB concentration for LAMP detection of each foodborne pathogen. The optimal HNB concentration was determined as 120 μM for *L. monocytogenes* (Figure S7A) and 90 μM for detecting the other three foodborne pathogens (Figures S7B-D).

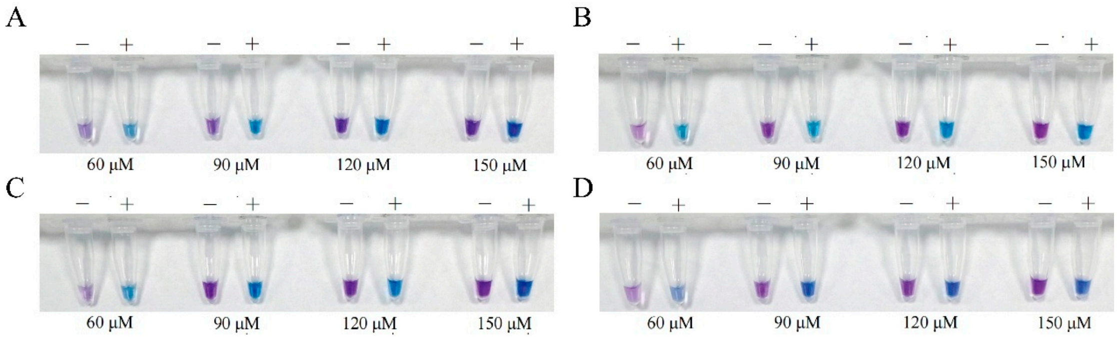

Figure S7. Optimization results of added concentration of HNB for LAMP detection of four pathogenic bacteria. The tube color results of LAMP amplification performed on *L. monocytogenes* (A), *S. aureus* (B), *S. enterica* (C), and *E. coli* O157:H7 (D) under different HNB concentrations. -: Negative reaction result, +: positive reaction result.

8. Culture-based analysis confirmed seven positive samples

On bismuth sulfite agar media, four *S. enterica* contaminated samples formed black-brown colonies with metallic sheen (Figure S8A-D), matching the morphology of *Salmonella* positive control (Figure S8E). Whereas, *E. coli* exhibited no visible growth on the medium (Figure S8F), confirming bismuth sulfite's inhibitory action against non-target bacteria.

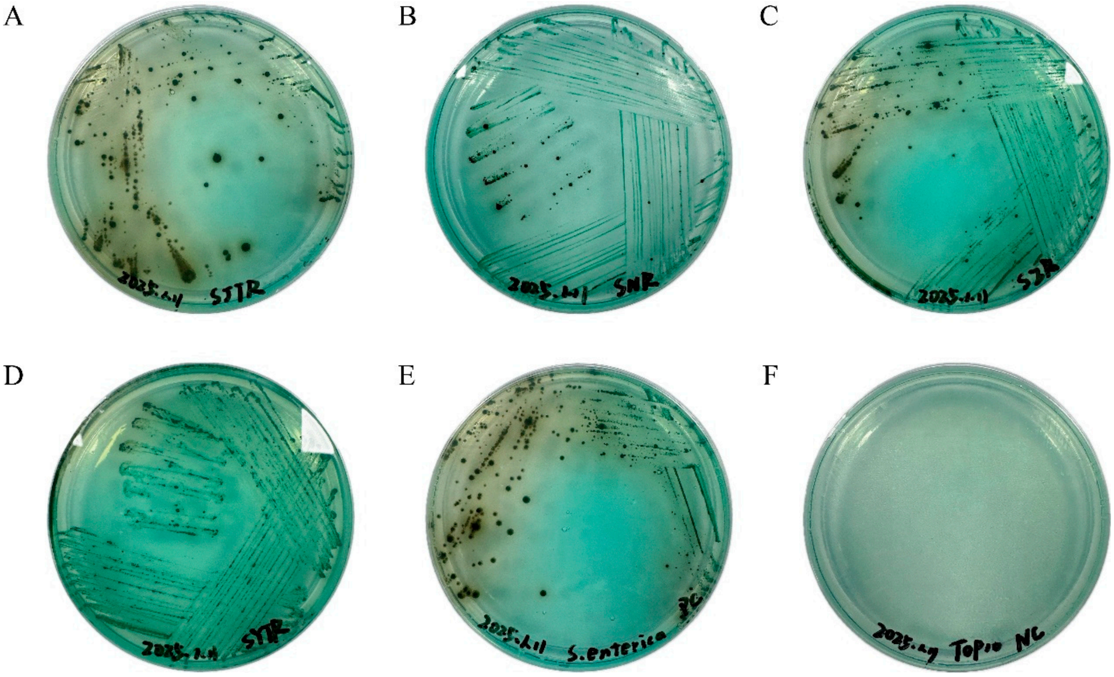

Figure S8. Microbial culture results confirming *Salmonella* positive samples. (A) raw chicken thigh meat, (B) raw beef, (C) raw pork, (D) raw duck thigh meat, (E) *S.enterica* as positive control, (F) *E.coli* as negative control.

On SMAC plates, three *E. coli* O157:H7 positive samples formed colorless and transparent colonies with smooth edges and a pale brown central region (Figure S9A-C), matching the morphology of *E. coli* O157:H7 positive control (Figure S9D). Whereas, *E. coli* served as the negative control, forming pink colonies on the medium (Figure S9E).

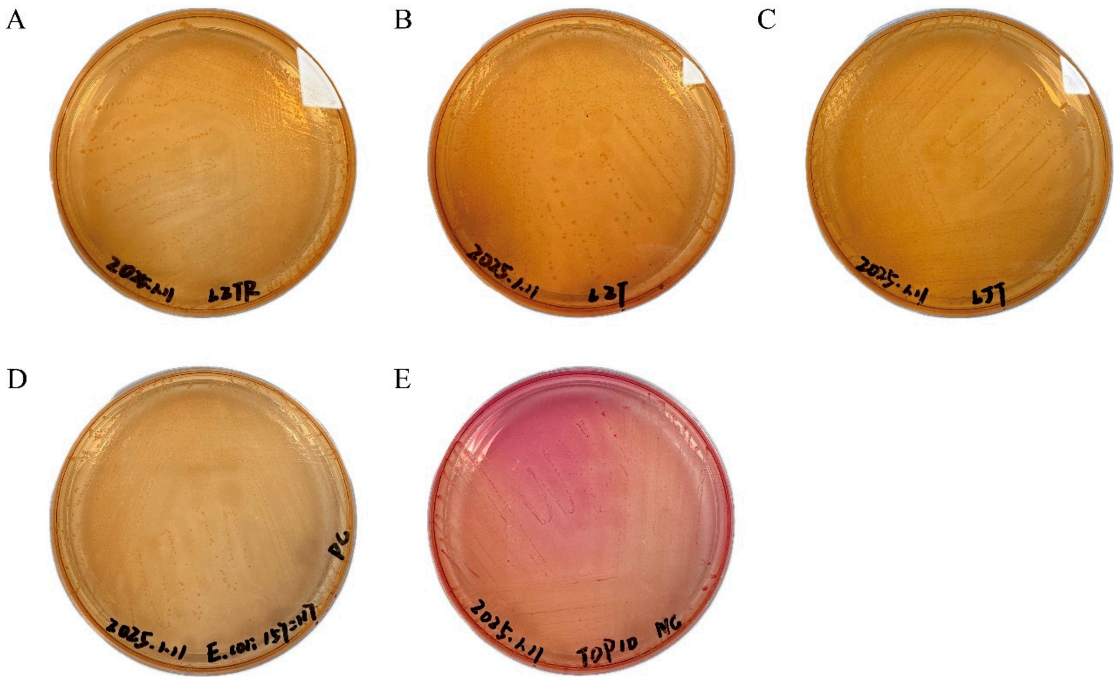

Figure S9. Microbial culture results confirming *E. coli* O157:H7 positive samples. (A) marinated pork head meat, (B) marinated chicken legs, (C) marinated pig trotters, (D) *E. coli* O157:H7 as positive control, (E) *E.coli* as negative control.
